# Kala-azar and Post–Kala-azar Dermal Leishmaniasis, Assam, India

**DOI:** 10.3201/eid2003.130260

**Published:** 2014-03

**Authors:** Abdul Mabood Khan, Prafulla Dutta, Siraj Ahmed Khan, Swaroop Kumar Baruah, Dina Raja, Kamal Khound, Jagadish Mahanta

**Affiliations:** Regional Medical Research Centre, Dibrugarh, India (A.M. Khan, P. Dutta, S.A. Khan, J. Mahanta);; Gauhati Medical College and Hospital, Guwahati, India (S.K. Baruah, D. Raja);; National Vector Borne Disease Control Programme, Guwahati (K. Khound)

**Keywords:** Visceral leishmaniasis, kala-azar, post–kala-azar dermal leishmaniasis, resurgence, Assam India, vector-borne infections, parasitic, parasites

**To the Editor:** Kala-azar (visceral leishmaniasis) is a fatal disease caused by a protozoan parasite *Leishmania donovani* and transmitted by the female sandfly, *Phlebotomus argentipes*. In the state of Assam, India, kala-azar epidemics occurred during 1875–1950 and resulted in thousands of deaths in the districts of Kamrup, Garo Hills, Goalpara, and Nagaon (*1*,*2*). The disease gradually disappeared from Assam because of the extensive use of DDT in the national malaria elimination program, and results of later entomologic studies indicated that there were no *P. argentipes *sandflies in this region after DDT use (*3*). However, sporadic kala-azar cases appeared again in Assam in 2004 (*4*), and in 2008, we reported a kala-azar outbreak in Kamrup (*5*), where kala-azar epidemics had occurred during the 1870s (*1*).

At bimonthly intervals during 2012, we conducted house-to-house surveys in 4 villages in the district of Kamrup, 845 households and 4,376 persons. Residents are socioeconomically poor and depend on agriculture and nearby brick kiln industries for their livelihood; persons involved in these industries generally keep cattle, and areas of cow manure provide breeding sites for sandflies. Persons reported with fever for >2 weeks, anemia, weight loss, and palpable spleen or liver and who were negative for malaria were tested for kala-azar by using the rK39 diagnostic kit (InBiOS, Seattle, WA, USA). We obtained bone marrow biopsy samples from selected persons who exhibited the symptoms listed above. A total of 162 persons had positive kala-azar results according to rK39 testing during 2008–2012; of these, 44 (27%) were children. Microscopic examination of bone marrow biopsy samples from 5 persons showed *L. donovani* parasites. We treated kala-azar case-patients with sodium stibogluconate (SSG). During the survey we recorded 4 suspected cases of post–kala-azar dermal leishmaniasis (PKDL).

Case-patient 1, a 16-year-old boy (panel A in Technical Appendix Figure ), was reported positive by rK39 for kala-azar in November 2008. After receiving 30 injections of SSG (20 mg/kg body weight), he became afebrile and his spleen decreased to a nonpalpable size. He gained weight, and hemoglobin improved to reference range. Three years after treatment, hypopigmented macules developed on his face, abdomen, and hands.

Case-patient 2 was an 18-year-old woman (Technical Appendix Figure, panel B). Kala-azar was diagnosed in 2011, and she received 30 injections of SSG. One 1 year after completing treatment, hypopigmented macules developed on her face and hands.

Case-patient 3 was a 16-year-old girl (Technical Appendix Figure, panel C). In 2008, after test results for kala-azar were positive, she received 30 SSG injections and clinically recovered. Macular hypopigmentation developed on her face and body 3.5 years after treatment.

Case-patient 4, a 45-year-old man (Technical Appendix Figure, panel D) was found positive for kala-azar in 2008 and received 17 doses of SSG. He had discontinued treatment because signs and symptoms subsided considerably, and he became afebrile.

Case-patients 1–4 were clinically examined to exclude other dermal diseases caused by fungi, vitiligo, and leprosy. These persons were also tested, and found to be negative, for tuberculosis, hepatitis C virus, and hepatitis B surface antigen. We obtained punched skin biopsy samples from each case-patient; a pinch of biopsy samples were dab smeared on glass slides for examination for .*L donovani* parasites, and remaining samples were stored in RNA*later* (QIAGEN, Hilden, Germany). We microscopically examined Giemsa-stained slides and found *L. donovani* parasite in 1 sample. Using QIAamp DNA Mini Kit (QIAGEN), we isolated parasite DNA from the samples and used it for the first round of PCR with primers 5′-AAATCGGCTCCGAGGCGGGAAAC-3′ and 5′-GGTACACTCTATCAGTAGCAC-3′ as described by Salotra et al. (*6*). Primers encompassing a 385-bp fragment internal to the 592-bp of *L. donovani* minicircle kinetoplast DNA having sequence 5′-TCGGACGTGTGTGGATATGGC-3′ and 5′-CCGATAATATAGTATCTCCCG-3′ (*7*) were used for nested PCR. Three samples were positive (Figure). We treated PKDL case-patients with amphotericin B deoxycholate in accordance with World Health Organization guidelines (*8*), and these patients recovered clinically.

**Figure:**
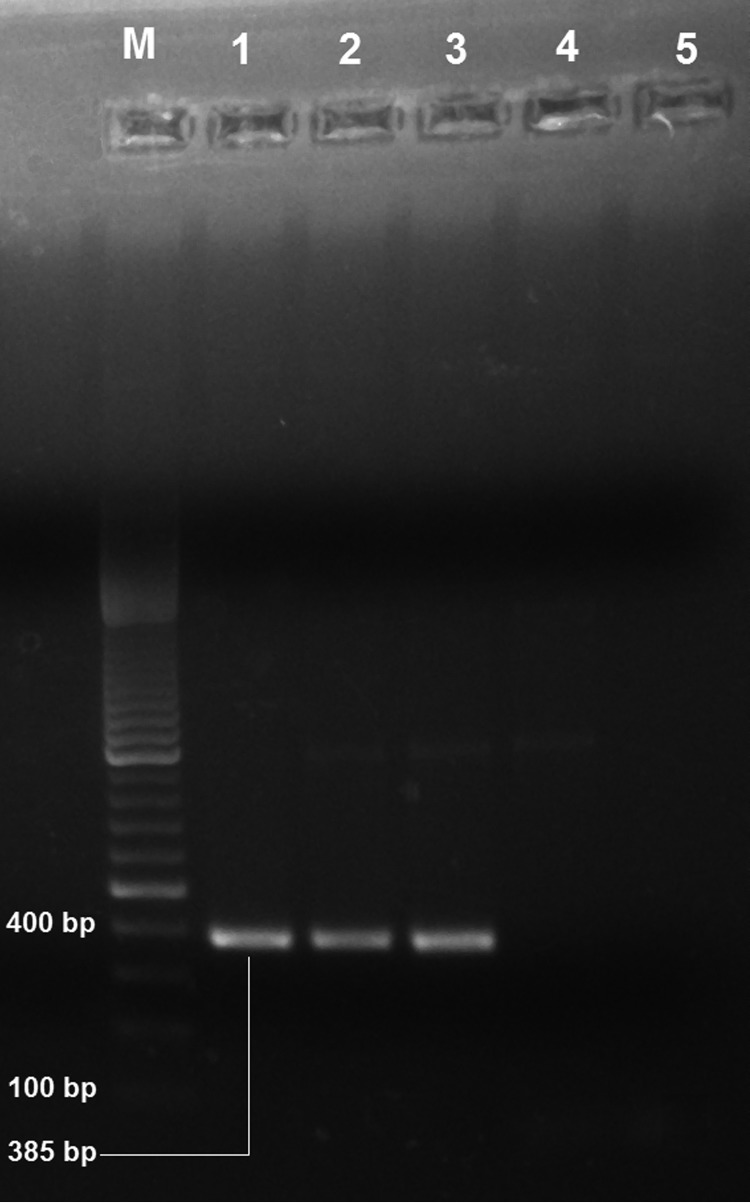
Electrophoretogram of *Leishmania donovani* kinetoplast DNA-specific PCR products (385 bp) isolated from patients with suspected post–kala-azar dermal leishmaniasis, Assam, India. Lane M, 100-bp DNA ladder; lanes 1–4, suspected post–kala-azar dermal leishmaniasis case-patients; lane 5, negative control. PCR products were visualized by staining with ethidium bromide after electrophoresis in 1% agarose gel.

Resurgence of kala-azar in the Kamrup district after a 60-year absence poses new challenges to India’s kala-azar elimination program. Of the 162 kala-azar cases detected, many were in children who had no history of visiting other kala-azar–endemic areas. These findings suggest local transmission of infection and are supported by the presence of the vector sandfly during the 2008 outbreak (*5*).

In India, PKDL develops in 5%–15% of treated kala-azar case-patients (*9*); in Sudan, conversion of kala-azar to PKDL is as high as 50% (*10*). PKDL cases act as reservoirs for kala-azar. Therefore, effective control depends on active surveillance for kala-azar and PKDL and treatment of kala-azar with antileishmanial drugs in accordance with Government of India guidelines (www.nvbdcp.gov.in/Doc/Guidelines-Diagnosis-Treatment-KA.pdf, www.nvbdcp.gov.in/Doc/PKDL-Guidelines-220512.pdf). Ecologic conditions of the areas where kala-azar outbreaks occurred are conducive to sandfly breeding; thus, regular spraying of DDT is needed. Preventive measures to control spread of kala-azar to other areas of Assam would be an effective step for the kala-azar control program.

Technical Appendix Skin lesions of patients with post–kala-azar dermal leishmaniasis, Assam, India.
