# Supplementary material for: Kala-azar and Post–Kala-azar Dermal Leishmaniasis, Assam, India
Source: Emerg Infect Dis. 2014 Mar;20(3):487–9. doi: 10.3201/eid2003.130260 (PMC3944866; doi:10.3201/eid2003.130260)
Supplement: Technical Appendix — Skin lesions of patients with post–kala-azar dermal leishmaniasis, Assam, India. [file 13-0260-Techapp-s1.pdf]

Article DOI: http://dx.doi.org/10.3201/eid2003.130260

Kala-azar and Post–Kala-azar Dermal Leishmaniasis, Assam, India

Technical Appendix

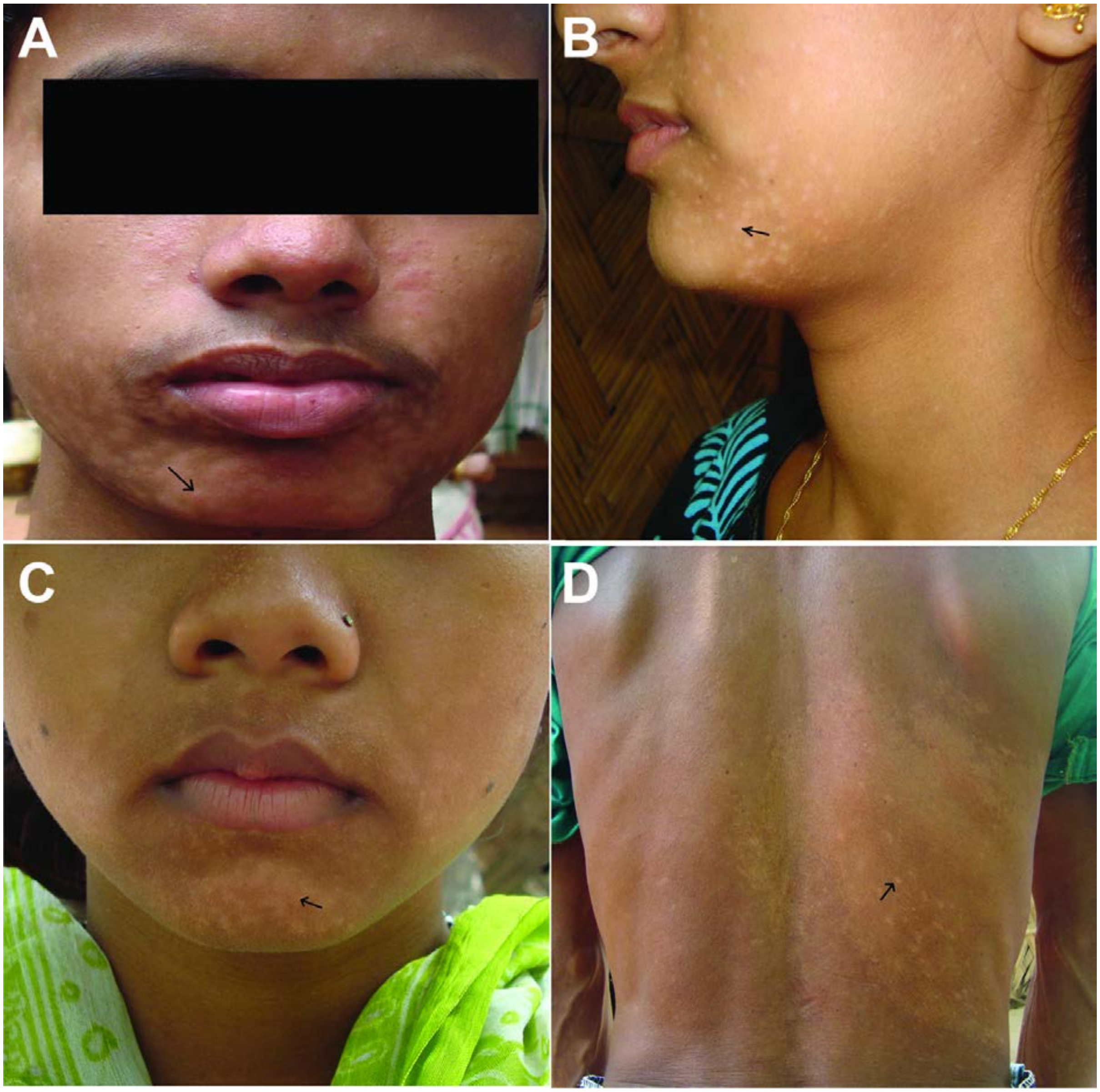

Technical Appendix Figure. Skin lesions of patients with post–kala-azar dermal leishmaniasis, Assam, India. A) 16-year-old boy. B) 18-year-old woman. C) 16-year-old girl. (D) 45-year-old man.
